# Intensive Environmental Surveillance Plan for *Listeria monocytogenes* in Food Producing Plants and Retail Stores of Central Italy: Prevalence and Genetic Diversity

**DOI:** 10.3390/foods10081944

**Published:** 2021-08-20

**Authors:** Gabriella Centorotola, Fabrizia Guidi, Guglielmo D’Aurizio, Romolo Salini, Marco Di Domenico, Donatella Ottaviani, Annalisa Petruzzelli, Stefano Fisichella, Anna Duranti, Franco Tonucci, Vicdalia Aniela Acciari, Marina Torresi, Francesco Pomilio, Giuliana Blasi

**Affiliations:** 1 Laboratorio Nazionale di Riferimento Per *Listeria monocytogenes*, Istituto Zooprofilattico Sperimentale dell’Abruzzo e del Molise G. Caporale, via Campo Boario, 64100 Teramo, Italy; g.centorotola@izs.it (G.C.); v.acciari@izs.it (V.A.A.); m.torresi@izs.it (M.T.); f.pomilio@izs.it (F.P.); 2 Istituto Zooprofilattico Sperimentale dell’Umbria e delle Marche “Togo Rosati”, via Gaetano Salvemini, 1, 06126 Perugia, Italy; d.ottaviani@izsum.it (D.O.); a.petruzzelli@izsum.it (A.P.); s.fisichella@izsum.it (S.F.); a.duranti@izsum.it (A.D.); f.tonucci@izsum.it (F.T.); g.blasi@izsum.it (G.B.); 3 ARS P.F. Prevenzione Veterinaria e Sicurezza Alimentare, Regione Marche, via Don Gioia, 8, 60122 Ancona, Italy; guglielmo.daurizio@regione.marche.it; 4 Centro Operativo Veterinario Per l’Epidemiologia, Programmazione, Informazione e Analisi del Rischio (COVEPI), National Reference Center for Veterinary Epidemiology, Istituto Zooprofilattico Sperimentale dell’Abruzzo e del Molise G. Caporale, via Campo Boario, 64100 Teramo, Italy; r.salini@izs.it; 5 Centro di Referenza Nazionale Per Sequenze Genomiche di Microrganismi Patogeni, Istituto Zooprofilattico Sperimentale dell’Abruzzo e del Molise G. Caporale, via Campo Boario, 64100 Teramo, Italy; m.didomenico@izs.it

**Keywords:** foodborne pathogen, food processing environments, monitoring plan, WGS typing, environmental stress resistance, QAC-resistance, persistence, virulence

## Abstract

*Listeria monocytogenes* (*Lm*) can persist in food processing environments (FPEs), surviving environmental stresses and disinfectants. We described an intensive environmental monitoring plan performed in Central Italy and involving food producing plants (FPPs) and retail grocery stores (RSs). The aim of the study was to provide a snapshot of the *Lm* circulation in different FPEs during a severe listeriosis outbreak, using whole genome sequencing (WGS) to investigate the genetic diversity of the *Lm* isolated, evaluating their virulence and stress resistance profiles. A total of 1217 samples were collected in 86 FPEs with 12.0% of positive surfaces at FPPs level and 7.5% at RSs level; 133 *Lm* isolates were typed by multilocus sequencing typing (MLST) and core genome MLST (cgMLST). Clonal complex (CC) 121 (25.6%), CC9 (22.6%), CC1 (11.3%), CC3 (10.5%), CC191 (4.5%), CC7 (4.5%) and CC31 (3.8%) were the most frequent MLST clones. Among the 26 cgMLST clusters obtained, 5 of them persisted after sanitization and were re-isolated during the follow-up sampling. All the CC121 harboured the *Tn6188*_*qac* gene for tolerance to benzalkonium chloride and the stress survival islet SSI-2. The CC3, CC7, CC9, CC31 and CC191 carried the SSI-1. All the CC9 and CC121 strains presented a premature stop codon in the *inlA* gene. In addition to the *Lm* Pathogenicity Island 1 (LIPI-1), CC1, CC3 and CC191 harboured the LIPI-3. The application of intensive environmental sampling plans for the detection and WGS analysis of *Lm* isolates could improve surveillance and early detection of outbreaks.

## 1. Introduction

*Listeria monocytogenes* (*Lm*) is a major foodborne pathogen causing human listeriosis, a severe zoonoses with high mortality. Invasive forms of the disease are particularly dangerous for the elderly, immuno-compromised people, newborns and pregnant women, leading to sepsis, meningitis, encephalitis, abortion and stillbirth [1]. Although listeriosis is relatively rare if compared with other foodborne disease, hospitalization (between 90% and 97%, for invasive forms) and fatality rates (approximately 20% to 30%, despite effective antibiotic treatment) make it a significant public health concern [2,3].

*Lm* is widespread in the natural environment, animals and food. Ready-to-eat products (RTE) (Table S1), such as salads or deli meat, are of special concern due to the lack of a heating step prior to consumption. Due to its ubiquity, the probability of introducing *Lm* into food producing plants (FPP), either with raw materials, through equipment or via employees is very high [4]. Therefore, it can be assumed that no FPP is *Lm* free [5]. Moreover, once introduced into a food processing environment (FPE), several factors increase the probability of a strain to establish long-lasting colonization of niches (harbourage sites) and to persist. Among them, there are the abilities of *Lm* to survive and grow under a wide range of environmental conditions (low pH, high salt concentration, alkaline and oxidative stress and refrigeration temperatures), to form biofilm and to show tolerance to common disinfectants used in the FPEs [4,6]. 

The European Commission Regulation 2073/2005 (Article 5) states that food business operators (FBO) manufacturing RTE, which may pose a *Lm* risk for public health, shall sample the processing areas and equipment for *Lm* as part of their Hazard Analysis and Critical Control Points plans. Such sampling activity aims at detecting *Lm*, including persistent strains, and at implementing corrective actions to eliminate the contamination. In Italy, except for the specific monitoring plan involving FPPs that are authorized to the export of meat products and other food of animal origin to the USA (DGISAN 42841-25 June 2019), a systematic and extensive monitoring of food surfaces is not included in the food safety surveillance programs performed by the FBO and competent authorities. 

Advanced molecular typing methods enable source attribution and investigation of *Lm* strains introduction and persistence in FPEs [7,8]. In particular, whole genome sequencing (WGS) allows an unprecedented subtyping resolution and is now considered the best typing tool in routine epidemiological surveillance of contamination. This method improves the detection of outbreaks, the understanding of distribution of virulent *Lm* strains in food and enables source attribution [9]. 

There is ample evidence of high variability regarding the virulence potential and pathogenicity of *Lm* isolates. Epidemiological data combined with results of WGS and from animal models, indicated that different levels of virulence may be associated with different clonal complexes (CCs). Maury et al. (2016) [10] distinguished CCs in ‘infection-associated’, ‘food-associated’ or ‘intermediate’ depending on the relative proportion of isolates from clinical cases, food or both. Clones CC1, CC2, CC4 and CC6 were strongly associated with human infection, whereas CC121 and CC9 were frequently isolated from food. Moreover, using a humanised mouse model, the authors observed that infection-associated clones were hyper-virulent, while the food-associated CCs were less invasive and hypo-virulent [10]. However, despite the observed variability in their virulence potential, almost every *Lm* strain has the ability to result in human listeriosis because of the complex interaction between the pathogen, food and the host [11]. 

Following a severe listeriosis outbreak occurred in Central Italy between 2015 and 2016 [12], with 24 confirmed clinical cases and associated with a pork-meat product, the Regional Competent Authority put in place an intensive environmental monitoring plan involving 86 FPEs in the affected area. The sampling plan was here described and was performed both at FPP and retail grocery stores (RS) level. Longitudinal follow-up sampling was conducted where positive samples were found, after cleaning and sanitation to verify their effectiveness and to give evidence of the elimination of the contamination by *Lm*.

The main objectives of the study were to: (i) provide a snapshot on *Lm* circulation in different FPP and RS, (ii) use WGS data to study the genetic diversity of the *Lm* isolates, identifying the most frequent and widespread clones and their virulence and stress resistance profiles, (iii) evaluate the genetic relationships between the isolates, identifying strains detected in more than one FBO or persisting despite sanitation.

## 2. Materials and Methods

### 2.1. Sampling and L. monocytogenes Detection

From January to August 2016, 1217 FPE surfaces were sampled from 41 FPP producing RTE and 45 RS located in Marche region, the samples were tested according to ISO 11290-1:1996/Adm1:2004 for *Lm* detection. In each FPE, a first sampling session was performed during processing and usually included 10 food-contact surfaces (FCS), as working tables, slicers, cutters, mixing and stuffing machines, containers, utensils, gloves, and 5 non-food-contact surfaces (NFCS), as floors, drains, sinks, walls, equipment framework, table legs, doors, boots, cleaning tools. If positive samples were found, the surfaces were tested again after extraordinary cleaning and sanitation (follow-up sampling). In accordance with the European Union Reference Laboratory for *Lm* (EURL) guidelines [13], the total sampled area varied depending on the sampling site, but was as large as possible to improve the probability of detecting *Lm*.

### 2.2. Statistical Analysis

All comparisons were made using a Bayesian approach with beta distribution by calculating 95% confidence intervals (CI 95%) (BETAINV function, Microsoft Excel, Redmond, WA, USA) for the percentages of positive sample. Differences between percentages were considered statistically significant when their CI 95% did not overlap.

### 2.3. Strains Collection

Up to five *Lm* colonies from one sample were randomly selected and screened for their belonging to one of the five major serogroups (IIa, IIb, IIc, IVa and IVb), using a multiplex PCR assay according to the EURL method [14,15]. One isolate for each serogroup found in each sample was submitted to WGS. 

### 2.4. Whole Genome Sequencing and Bioinformatics Analysis

DNA extraction was performed using the Maxwell 16 tissue DNA purification kit (Promega Italia Srl, Milan, Italy) according to the manufacturer’s protocol and the purity of the extracts was evaluated by NanoDrop2000 (ThermoFisher Scientific, Wältham, MA, USA). Starting from 1 ng of input DNA, the Nextera XT DNA chemistry (Illumina, San Diego, CA, USA) for library preparation was used according to the manufacturer’s protocols. WGS was performed on the NextSeq 500 platform (Illumina, San Diego, CA, USA) with the NextSeq 500/550 mid output reagent cartridge v2 (300 cycles, standard 150-bp paired-end reads).

For the analysis of WGS data, an in-house pipeline [16] was used which included steps for trimming (Trimmomatic v0.36) (base quality parameters—Leading: 25; Trailing: 25; Slidingwindow: 20:25) [17] and quality control check of the reads (FastQC v0.11.5). Genome de novo assembly of paired-end reads was performed using SPAdes v3.11.1 [18] with the parameters suggested by the manual for the Illumina platform 2 × 150 chemistry (--only-assembler --careful -k 21, 33, 55, 77). Then, the genome assembly quality check was performed with QUAST v.4.3 [19]. 

The genome assemblies were deposited at DDBJ/ENA/GenBank under the BioProject PRJNA737760.

The MLST scheme used to characterize *Lm* strains is based on the sequence analysis of seven housekeeping genes (*abcZ*, *bglA*, *cat*, *dapE*, *dat*, *ldh and lhlA*) [20].

The seven-gene of MLST scheme and the clonal complexes (CCs) were deducted in silico using the BIGSdb-*Lm* database (http://bigsdb.pasteur.fr/listeria; accessed on 14 April 2021).

For the cluster analysis of the strains, the core genome MLST (cgMLST), according to the Institut Pasteur’s scheme of 1748 target loci, was performed using the chewBBACA allele calling algorithm [21], available in the in-house pipeline. Agreeing to the guidelines for *Lm* cgMLST typing [22], only the genomes with at least 1660 called loci (95% of the full scheme) were considered. Using the software GrapeTree [23] a Minimum Spanning tree (MSTreeV2 method), showing the relationships among the strains in terms of allelic mismatches was generated. 

The genomes of the strains belonging to the most frequently isolated CCs, for which at least five isolates were detected, were further characterized using “M*etal and detergent resistance genes*”, “*Stress Islands*” and “*Virulence*” tools of the BIGSdb-*Lm* database (accessed on 18 May 2021). The gene presence/absence matrices according to their MSTree were visualized using Phandango v 1.3.0 [24] (accessed on 30 July 2021).

## 3. Results

### 3.1. Sampling and L. monocytogenes Detection

A total of 1217 samples were collected in the first sampling session. Forty-six out of the 86 different establishments showed at least one positive sample and were considered contaminated by *Lm*. The percentage of positive facilities was 60.9 % (CI 95%: 45.6–74.4%) at FPP and 46.7% (CI 95%: 32.9–61.0%) at RS.

A total of 118 samples (9.7%; CI 95%: 8.2%–11.5%) were positive for *Lm*, of which there were 72 (12.0%; CI 95%: 9.6–14.8%) at FPP and 46 (7.5%; CI 95%: 5.7–9.8%) at RS. No statistically significant difference was found in the amount of *Lm* positive samples between these two types of FBO. At single establishment level, the percentage of positive samples ranged between 7% and 80% at FPP and from 7% to 40% at RS.

Overall, *Lm* was detected in 76 (9.6%; CI 95%: 7.8–11.9%) FCS and 42 (9.8%; CI 95%: 7.3–13.0%) NFCS. The difference between positive FCS at FPP (12.9%; CI 95 %: 9.9–16.7%) and at RS (6.6%; CI 95%: 4.6–9.4%) was found to be significant (Table 1).

In order to identify differences in prevalence, both FCS and NFCS were grouped into five categories after sampling: equipment (e.g., working tables, containers, hooking bars, cutting boards), industrial systems (e.g., floors, walls, drains, sinks, door handles, freezers), machines (e.g., slicers, blenders, mincers, hoppers, labellers), clothing (e.g., shoes and gloves) and tools (e.g., knives, ladles and tongs) (Table S2).

In Table 2 the results of FCS and NFCS are showed according to the category they belonged to.

Not including the clothing category, for which the samples number was small when compared with the others, the FCS of the equipment appeared the most contaminated. The NFCS presented the highest number of positive samples in the industrial system category (Table 2). These differences were not statistically significant.

At RS, the percentage of positive surfaces belonging to industrial systems, machines and tools was lower than what was observed in FPP (Table 3).

A follow-up sampling session was carried out in 33 out of the 46 FPP that tested positive, after cleaning and sanitation. Positive surfaces were still found in 2 FPP and 2 RS. They were re-sampled and re-analysed until they resulted as negative for the presence of *Lm*. During these follow-up activities, a total of 279 samples were collected and 11 of them tested positive. 

### 3.2. Strains Collection

A total of 133 *Lm* strains, 121 isolated within the first sampling session and 12 during the follow-up activities, were selected and collected to be typed (Tables S3 and S4). Eighty isolates were from FPP and 53 from RS. In Table S5, all the *Lm* strains of the study were reported. Among all the *Lm* strains analysed, four serogroups (IIa, IIb, IIc, and IVb) were identified. The main was the serogroup IIa revealed for 55 strains (28 RSs, 27 FPPs), followed by IIc for 30 strains (26 FPPs, 4 RSs), IIb for 27 strains (20 FPPs, 7 RSs) and finally, serogroup IVb revealed for 21 strains (15 FRs, 6 FPPs). In 11 plants, both from FPP and RS, at least two different serogroups were detected.

### 3.3. WGS and Bioinformatics Analysis

For all the 133 genomes, sequence data were obtained in agreement with the quality control thresholds recommended. Quality metrics of sequence data obtained for each genome are reported in Table S5. 

#### 3.3.1. Distribution of CCs and cg-MLST Clusters

**Figure 1:**
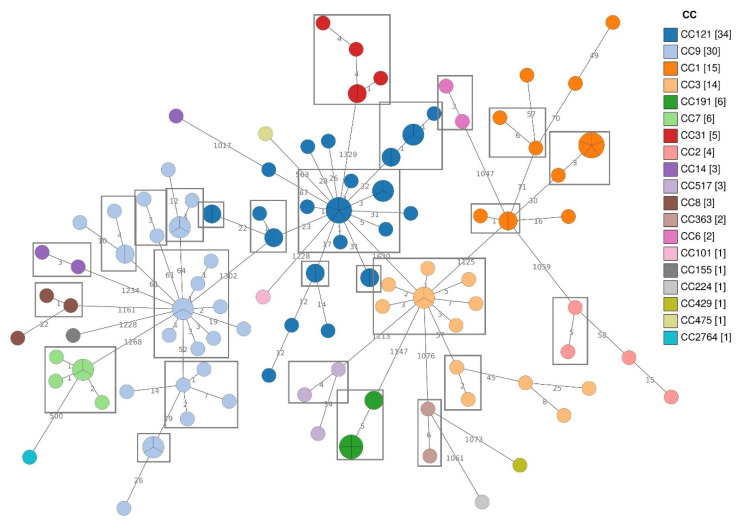
Minimum Spanning Tree (MST) based on the cgMLST profiles of 133 *Lm* strains, coloured according to CCs.

MLST analysis grouped the strains in 19 CCs (Table S3, Figure 1). More in detail, the *Lm* strains belonged to the following CCs: CC121 (25.6%), CC9 (22.6%), CC1 (11.3%), CC3 (10.5%), CC191 (4.5%), CC7 (4.5%), CC31 (3.8%), CC2 (3.0%), CC517 (2.3%), CC8 (2.3%), CC14 (2.3%), CC363 (1.5%), CC6 (1.5%), CC155 (0.8%), CC224 (0.8%), CC429 (0.8%), CC475 (0.8%), CC101 (0.8%). In one strain, isolated in the retail plant RS9, exact allele matches were found only for five of the seven genes of the MLST scheme. The genome of this strain was submitted to the BIGSdb-*Lm* database to be typed and its MLST profile resulted new in the database (submitted on 13 August 2021). New alleles and profiles were defined with the assignation of CC2764 (Figure 1 and Table S3). 

Among the CCs isolated in both the FBO types, CC121, CC9 and CC3 presented most of the isolates from FPP, while CC1, CC7 and CC14 from RS. *Lm* strains belonging to CC191, CC17, CC6 and CC429 were found only in FPP, while CC31, CC2, CC8, CC36, CC101, CC155, CC224 and CC475 were exclusively isolated in RS. 

The cgMLST cluster analysis was performed to deepen the relationships among the *Lm* isolates (Figure 1). According to the cgMLST allelic threshold (≤7) for cluster definition [22], 26 cgMLST clusters were identified among all the isolates. Strains belonging to CC121, CC9, CC1 and CC3 grouped into more than one cluster (Table 4).

All the CC2, CC6, CC7, CC8, CC14, CC31, CC191 and CC363 strains presented a single cgMLST cluster with CC2, CC8 and CC14 also including some singletons. The remaining CCs presented only singleton strains (Figure 1).

Several CCs and cgMLST clusters were isolated from different sampling points, both in FPP and RS (Table S3). Five cgMLST clusters belonging to CC7 (1), CC9 (2) and CC121 (2), were detected at different time points, both during the first sampling and the follow up control, in the same FPE (Figures S1–S5). The FPEs in which a specific cgMLST cluster was re-isolated after sanitation were 4: two FPPs and two RSs.

More in detail, CC121 isolates (Figure S1) were from six FPPs and six RSs. For this CC, none of the cgMLST clusters found was shared by more than one FPE (Figure S1). 

*Lm* strains belonging to CC9 were isolated in 10 FPPs and 2 RSs. Two CC9 cgMLST clusters were detected in more than one FBO (Figure S2). 

CC1 strains were isolated in five FPPs and four RSs. (Figure S3). 

All CC3 strains were isolated during the first sampling session from FPP2, FPP22, FPP25, RS4, RS14 and RS17 (Table S3). Strains belonging to the same cgMLST cluster were detected in RS4, FPP2 and RS17 (Figure S4). 

The CC191 clone was represented by six strains, all isolated from FPP1 and belonging to the same cgMLST cluster.

All the CC7 strains grouped in the same cluster isolated in RS10, RS13, RS14 and FPP4 (Tables S3 and S4, Figure S5). 

CC31 was exclusively isolated from RSs and consisted of five strains, isolated in five different RSs (RS12, RS18, RS19, RS20 and RS21) (Table S3) and belonging to the same cgMLST cluster. 

Strains belonging to CC2 were collected from RS5, RS8 and RS13 (Table S3). Only two of them, both isolated from RS8 during the first sampling session, belonged to the same cluster.

Three CC8 were isolated from RS4 and RS7 during the first sampling session (Table S3). Allelic differences ≤7 were found only in two strains belonging to this CC, both isolated in the RS4 exercise. 

Three CC14 *Lm* were found in this study and they were collected from FPP18 and RS6 (Tables S3 and S4). Both the strains from the RS6 isolated during the first and the follow-up sampling session, respectively, belonged to the same cluster.

For the remaining CCs, only one strain was isolated during the study.

#### 3.3.2. Detection of Stress Resistance and Virulence Genes

CC1, CC3, CC7, CC9, CC31, CC121 and CC191 were considered the most frequently detected CC as for each of them at least five *Lm* were isolated. Strains belonging to these CCs, 110 in all, were further characterized.

**Figure 2:**
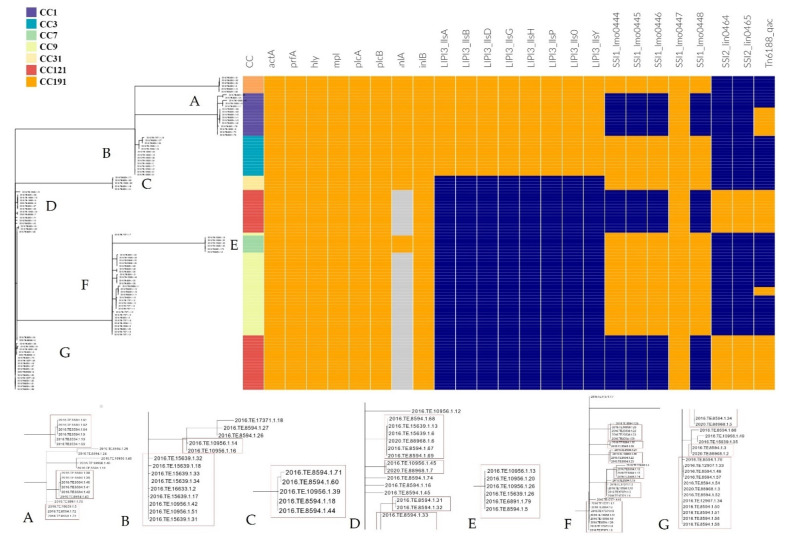
Stress resistance and virulence patterns according to CCs and cgMLST clustering. Orange: presence of the gene; light blue: presence of premature stop codon; blue: absence of the gene. Enlarged detail images of different nodes are shown at the bottom of the figure and are indicated with the letters from (**A**–**G**). The red boxes enclose the isolates belonging to the same cgMLST cluster. The incomplete cluster of image D is completed by the one in the image (**G**).

The in silico results on presence/absence of disinfectants resistance genes, SSIs and virulence genes (Figure 2) showed that all the CC121 strains harboured the *Tn6188*_qac for tolerance to benzalkonium chloride (BC). This gene was also detected in 10 CC1 (66.7%) and in three CC9 (10.0%) strains.

All the strains belonging to CC9 CC3, CC191, CC7 and CC31 carried out the five genes of SSI-1 (*lmo*0444, *lmo*0445, *lmo*0446, *lmo*0447 and *lmo*0448), while in CC121 and CC1, only *lmo*0447 gene was found. Moreover, the two genes of SSI-2 (*lin*0464 and *lin*0465) were only detected in CC121 strains.

Regarding the virulence genes, all CC1, CC3, CC191, CC7 and CC31 strains carried a full length *inl*A for Internalin A and *inl*B for Internalin B. A Premature Stop Codon Mutation (PMSC) in the *inl*A gene was detected in all the CC121 and the CC9 strains. 

All the studied CCs presented *prf*A, *plc*A, *hly*, *mpl*, *act*A and *plc*B, forming together the *Lm* Pathogenicity Island 1 (LIPI-1). Moreover, CC1, CC3 and CC191 strains also harboured a complete LIPI-3 composed of the eight *lls* genes (*lls*A, *lls*G, *lls*H, *lls*X, *lls*B, *lls*Y, *lls*D, *lls*P).

## 4. Discussion

This retrospective study reported the results of an intensive environmental monitoring plan carried out during the investigation tracing steps of a severe listeriosis outbreak that occurred in Central Italy between 2015 and 2016 [12]. The results provided information about the environmental contamination of *Lm* circulating in pork-meat FPP and RS of Marche Region. 

### 4.1. Sampling and L. monocytogenes Detection

The importance of the study was to give evidence of the distribution and diversity of *Lm* strains, as in previously studies carried out on FPP and in RS [25,26,27,28]. We collected surfaces samples, including both FCS and NFCS, from 41 ready-to-eat pork-meat FPPs and 45 RSs of the studied area and tested them for *Lm* detection. 

We found that 60.9% of FPPs and 46.7% of RSs were contaminated by *Lm*. A total of 72 (12%) samples were positive for *Lm* at FPP and 46 (7.5%) at RS levels. Results showed no significant differences for FPP and RS, surfaces were widely positive for *Lm* and possible sources of food contamination. 

Antoci et al. (2021) reported in their study that 50% of the FPPs were contaminated by *Lm*, with at least one sample positive for the pathogen [29]. They also reported that 9.8% of FCS and 6.1% of NFCS were contaminated by *Lm*. We found a higher percentage of contaminated FPPs, all located in the same Region of Italy. Even the percentage of positive FCS found at the FPPs was higher in our study (12.9%) and it was probably due to the high levels of *Lm* contamination existing in raw pork [30,31]. 

Antoci et al. (2021), in fact, included in their monitoring plan also FPPs for meat, fishery and dairy products, while in the present study all the FPPs were in the pork chain [29]. All these findings suggested a massive spread of *Lm* in the pork production chain of Marche Region, emphasizing the need for more assiduous monitoring and more effective risk containment measures to prevent food contamination.

As reported by Forauer et al. (2021), many studies in this field were carried out in the U.S.A [32]. Hoelzer et al. (2011) and Sauders et al. (2009), in their non-longitudinal deli-focused studies conducted in RS of the New York State, reported that approximately 60% of them were positive for *Lm* [26,33]. Etter et al. (2017) [34], in a recent longitudinal study in 30 U.S.A. delis, found that about 97% of them tested positive for *Lm* at least once, while Burnett et al. (2020) [27] monitored 30 grocery stores across seven U.S.A states, finding that *Lm* was isolated at least once from 83% of them. All these results showed higher percentages of RSs contaminated by *Lm* than the level reported in our study, probably linked to the larger geographical area involved in the U.S.A studies.

The obtained results showed how FCS and NFCS could equally harbour *Lm*, representing sources of food contamination or possible persistence niches. These findings emphasized the importance of sanitation procedures including effective strategies to clean and sanitize NFCS. More in detail 12.9% FCS and 10.4% NFCS tested positive at FPP, while 6.6% FCS and 9.2% NFCS at RS.

The difference between FCS and NFCS was not significant, even within each type of FPE tested. This result was not in line with previous studies reporting that *Lm* prevalence was significantly lower on FCS than on NFCS and indicated a widespread of *Lm* in the studied FPEs [26,34,35].

The number of positive FCS at FPPs level was significantly higher than at RS level. This result could be explained with the large amount of raw material handled in FPPs also considering that high levels of *Lm* contamination in raw pork have been regularly reported [30,31].

All the surfaces’ categories, including both FCS and NFCS sampled (equipment, industrial systems, machines, etc.), reported positive results for *Lm* indicating the need to include them all in the monitoring plans. The lack of statistical significance resulting from comparisons between these categories (FCS vs. NFCS; FPP vs. RS) was in part due to the different sample size as we grouped surfaces only after sampling to further investigate the prevalence of *Lm* contamination. 

During the follow-up sampling session in two FPPs and two RSs, FCS previously resulted positive for *Lm*, were found contaminated again despite sanitation. The persistence of *Lm* contamination on these surfaces could be explained by the ineffectiveness of cleaning and sanitation procedures used, the incorrect application or specific stress-resistance abilities of the contaminating *Lm* strains. 

A limitation of this work was that, being a retrospective study performed years after the emergency when the monitoring plan was finished, it cannot give information about sources of *Lm* contamination and transmission routes. 

### 4.2. Distribution of CCs and cg-MLST Clusters and Their Virulence and Stress Resistance Profiles

*Lm* serogrouping is considered a first typing step useful to evaluate the microbial population diversity. The serogroup IIa was reported as the most frequently isolated [36,37], in agreement with our results (Table S3) and other studies conducted in meat products and environmental surfaces [35,38,39,40].

To have more insight regarding the spread of *Lm* in different FPE, WGS was performed to analyse the diversity of the *Lm* strains detected during the study, identifying genetic relationships between the strains and detecting virulence and stress resistance associated determinants. The MLST and the cgMLST analysis showed a great heterogeneity of the *Lm* population circulating in the studied area, identifying 19 different CCs and 26 cgMLST clusters. The MLST clones most frequently isolated in this study (Figure 1; Table S3), were already defined as the most frequent clones in many countries [39,41,42].

CC1, CC3, CC9 and CC121 presented the greatest genetic diversity, in terms of cgMLST clusters. 

With the exception of CC31 and CC191, isolated only at RS and FPP, respectively, all the CCs were found in both type of FPE. In some cases, it was just the same cgMLST cluster to be isolated in different FPP and RS FPEs. 

According to previous studies, the CC9 and CC121 were considered hypo-virulent clones, able to cause disease in highly immune-compromised individuals and seeming to be better adapted to FPE and presenting strong association with the meat processing environment [6,39,41]. Several authors also reported that CC9 and CC121 presented a higher prevalence of stress resistance and BC tolerance genes, a higher survival and biofilm formation ability and were able to persist in FPE even for years [6,41]. Moreover, in a recently published study, Guidi et al. (2021) reported two different CC9 clusters persisting, for four and two years, respectively, in a pork-meat processing plant of the same studied area of central Italy [6]. All these findings were consistent with our results and in particular with the isolation and persistence of these CCs after sanitation. Indeed, most of the strains isolated both during the first sampling and the follow-up in the same FPE belonged to CC121 and CC9, harbouring the *Tn6188*_qac transposon for tolerance to BC, a quaternary ammonium compound widely used in food industry. The SSIs are known to confer resistance to stresses, in particular the SSI-1, linked to environmental stress, such as low pH, high osmolarity, bile and nisin, and the SSI-2, linked to tolerance to alkaline and oxidative stresses. According to our results, SSI-1 were frequently observed in *Lm* strains belonging to different clones, whereas the SSI-2 genes were mainly found in CC121 isolates [43], suggesting a possible contribution to strains adaptation and persistence in FPPs [44].

In previous studies the CC1 clone was isolated in the pork-meat production and in other production sectors although it was more abundant and strongly associated with milk and the dairy sector [45]. Moreover, CC1 was previously defined hyper-virulent with a high clinical frequency [10,41]. For this reason, its spread in the FPE of the studied area should be taken into consideration, for the risk of cross-contamination between surfaces and food. 

The clone CC3 was one of the most prevalent in cooked products according to Wang et al. (2018) [46], while a recent study reported it was over-represented in the RTE of poultry origin and in meat FPPs [47].

CC7 isolates were previously globally recovered (North and South America, Europe, Oceania, Africa and Asia) from a variety of sources, such as wild animals, ruminants, poultry, silage, fish, slaughterhouse floors, compost and human infections [48]. A high prevalence of CC7 at the dairy farm level in the USA was also reported [49]. Among the CC7 strains isolated during this monitoring plan, four belonged to the same genetic cluster (analysis results not shown) causing the severe invasive listeriosis outbreak reported by Duranti et al. (2018) and occurred between 2015 and 2016 in the studied area [12]. This cluster, never detected before in any of the studied FPEs, were recovered in one FPP and two different RS, showing how the *Lm* outbreak strain was widely circulating in the FPE of central Italy. Moreover, this clone re-emerged in the same area during 2018, when it was isolated from a child affected by listeriosis, presenting only 13 single-nucleotide polymorphisms (SNPs) of difference from the original outbreak strain [50]. All these findings emphasized the need for continuous monitoring in order to avoid the recurrence of new listeriosis outbreaks.

The CC191 clone was poorly reported in the literature. Recently, Kurpas et al. (2020) included in their study a CC191 strain isolated from a slaughterhouse [51]. 

Maury et al. (2016) [10] observed a strong association of CC31 with meat and meat products, as reported also by the European Food Safety Authority [7]. The spread of CC31 in FPE of FPP of the meat chain, in agreement with our results, was also confirmed by a recent published study, reporting CC31 isolates from environmental samples collected in meat FPP and farms [47]. 

The other CCs presenting less than five isolates were mostly linked to the RS and included both hypo- and hyper-virulent clones. Among them, CC2 was previously defined as a hyper-virulent infection-associated clone as the CC1, described above, and the CC6. Maury et al. (2019) evaluating the CC proportion in different food categories, found CC2 isolates from different food groups without a strong association with anyone in particular [41]. Recently, Guidi et al. (2021) reported CC2 strains persisting over four years in a dairy facility both at food and environmental levels [6].

According to several authors, the other remaining CCs detected in this study were previously isolated from different sources, such as food and environments [10,45,46,48,52,53,54,55,56,57]. 

Within each FPE we found different degree of diversity both at CCs and cgMLST clusters level. More in detail within the FPPs studied, a maximum of two different CCs and three cgMLST clusters were detected, while at the RS level, up to five different CCs were isolated in the same FPE, although a less strain variability within each CC was observed. The greater genetic diversity observed at RS level was most likely related to the high variability of food products and food categories, from different suppliers, handled at RS level. 

Only five cgMLST clusters were detected both at the first sampling and at the follow up control in four different FPEs (Figures S1–S5). Four of five clusters, two belonging to CC121, one to CC7 and one to CC9, included strains with 0–1 allelic differences, therefore their re-isolation after cleaning and sanitation could be considered as due to persistence of the same strain. The last cgMLST cluster (the second cluster of CC9), instead, was composed by two isolates with five alleles difference, in this case the hypothesis of a reintroduction could not be excluded.

Very interestingly, the CC7 and the two CC9 cgMLST recurrent clusters detected after sanitation in the same FPE (RS13) were not carriers of the Tn6188_*qacH* gene, specific for BC resistance. The lack of specific determinants for tolerance to sanitizers and the re-isolation of three different cgMLST types after sanitation in the same FPE, suggested that cleaning and sanitation protocols used were ineffective. In contrast, all the CC121 strains grouping in the same cgMLST clusters were carriers of the Tn6188_*qacH* gene and their isolation at different time points could be due to their resistance to sanitation. Although we do not have detailed information regarding the specific disinfectants used in each FPE, it is known that QAC and specifically BC, are the most commonly used in the food industry. 

From all these findings several recommendations were provided to FBOs in order to remove or reduce resident *Lm*, such as the use of different sanitizers combining or turning them and the application of procedures needed to clean and disinfect niches or harbourage points.

Virulence factor analysis was performed on seven CCs (CC1, CC3, CC7, CC9, CC31, CC121 and CC191) presenting at least five isolates and considered widespread in the studied area (Figure 2). 

As previously reported, among the major virulence factors crucial for the intracellular lifestyle of *Lm* there is the LIPI-1, highly conserved among *Lm* strains and containing the *prfA*, *plcA*, *hly*, *mpl*, *ActA* and *plcB* genes [36,58]. As expected, LIPI-1 was detected in all the strains.

All CC1, CC3, CC7 and CC191 isolates carried a full-length *inl*A and *inl*B, considered one of the most influent factors on the *Lm* invasiveness [58]. Internalins are surface proteins used by *Lm* to invade and cross the human intestinal barriers invading epithelial cells during the infection process and among them Internalin A (InlA) and B (InlB) are considered the most relevant [58,59,60]. These findings confirmed CC1 as hyper-virulent clone, as reported before [10,41] and suggested the same for CC3, CC191 and also for CC7, to which belonged the outbreak strain described by Duranti et al. (2018) [12]. On the contrary, a PMSC in the *inl*A gene, mainly detected in all the CC121 and the CC9 strains, confirmed these clones as hypo-virulent. 

Moreover, according to previous reports [36], all the CC1, CC3 and CC191 also harboured a complete LIPI-3, encoding a biosynthetic cluster involved in the production of Listeriolysin S (LLS) [61]. LLS (hemolytic and cytotoxic factor conferring a greater virulence to *Lm*) is expressed only under oxidative stress conditions and this confers a better ability in terms of phagosome escape. Therefore, the presence of LIPI-3 is considered responsible for the increased virulence in some strains [61,62].

## 5. Conclusions

The present study represented the first intensive *Lm* FPE monitoring plan performed in central Italy, both at FPP and RS level, within the pork-meat chain. Results highlighted that FPEs widely harboured *Lm*, both on FCS and NFCS, representing potential sources of food cross contamination. A systematic *Lm* monitoring of FPEs in Italian food safety surveillance plans performed by the competent authority should be included, designing an effective, risk-based environmental monitoring program, and defining the guidelines for key design elements, such as the number, location, timing and frequency of sampling as well as standard criteria for classifying surfaces into specific categories. Moreover, there are no common standard criteria to classify surfaces into a specific category. Therefore, it should be very important to define a standard categorisation of food surfaces to be used in monitoring plans in order to obtain comparable results. However, these recommendations should take into account that each FPE has specific characteristics and different critical points and so provide for flexible and adaptable criteria to each food associated reality. Moreover, sinks and drains (NFCS) should not be excluded as they were very often contaminated with *Lm*. The *Lm* circulation in FPEs, with a common presence of strains at FPP and RS level after the follow-up sampling, should focus the attentions at the efficacy of cleaning and disinfection procedures. 

Thanks to the highly discriminatory power, WGS is now routinely used for the surveillance of human listeriosis and for food-safety monitoring. The great benefit and the potential of WGS analysis emerged from this study, emphasizing how this advanced molecular typing method should also be considered an essential tool in the environmental monitoring plans. Moreover, WGS could also easily detect the possible presence of different *Lm* clones and clusters within the same *Lm* positive sample, showing how is extremely important trying to isolate more than one strain from each positive sample analysed. 

Through the cgMLST cluster analysis, the genetic relationships between isolates were investigated allowing us to identify strains persisting after sanitization in the same FPE as well as strains contaminating different FPEs. The spread, both at FPP and RS, of hypo-virulent CCs, more adapted to FPEs and able to persist after cleaning and sanitation represents a significant risk of food cross contamination. On the other hand, the detection of hyper-virulent clones, including an outbreak strain, even without evidence of persistence, posed an even more warring risk for the public health. 

The provided information contributed to increasing knowledge on the environmental spread of *Lm* in meat FPP and RS of Marche Region, following a severe listeriosis outbreak occurred between 2015 and 2016. The lack of European environmental monitoring studies including both FPP and RS and in particular the paucity of data on *Lm* FPE contamination at RS, emphasize the need to add FPEs to the sampling plan and collect data on the topic in this continent. From the results obtained in this study arose several recommendations to be provided to FBOs and aimed at improving the management of the pathogen minimizing risk of food contamination and recurrence of severe outbreak of listeriosis.

In conclusion, the application of intensive environmental sampling plans, considering several different surfaces, for the *Lm* detection and the isolation, when possible, of more than one *Lm* strain from each positive sample might be extremely important, in order to have improved surveillance, better clusters detection and early foodborne outbreak detection.

## Figures and Tables

**Table 1:** Results of tested surfaces based on the food environment typology.

Surfaces	FCS				NFCS			
Food Producing Environments	*n*	*Lm*+	%	CI 95%	*n*	*Lm*+	%	CI 95%
FPP	379	49	12.9	9.9–16.7	222	23	10.4	7.0–15.1
RS	409	27	6.6	4.6–9.4	207	19	9.2	6.0–13.9
Total	788	76	9.6	7.8–11.9	429	42	9.8	7.3–13.0

FPP: food processing plant; RS: retail store; FCS: food contact surfaces; NFCS: non-food contact surfaces; *Lm+*: *Listeria monocytogenes* positive samples.

**Table 2:** Results of the tested FPEs based on the category.

	FCS				NFCS			
Surface Category	*n*	*Lm*+	%	CI 95%	*n*	*Lm*+	%	CI 95%
Equipment	346	39	11.3	8.4–15.0	83	6	7.2	3.4–14.9
Industrial systems	64	5	7.8	3.4–17.0	267	29	10.9	7.7–15.2
Machines	259	20	7.7	5.0–11.6	64	4	6.3	2.5–15.0
Clothing	5	1	20.0	3.6–62.5	9	3	33.3	12.1–64.6
Cleaning tools	0	0	0	0	2	0	0	0
Tools	114	11	9.6	5.5–16.5	3	0	0	0
Not classifiable surface	0	0	0	0	1	0	0	0
Total	788	76	9.6	7.8–11.9	429	42	9.8	7.3–12.9

FCS: food contact surfaces; NFCS: non-food contact surfaces; *Lm+*: *Listeria monocytogenes* positive samples.

**Table 3:** Results of the samples tested reported based on the FPP or RS and category of surfaces (*n* = 1214 *).

	FPP				RS			
Surface Category	*n*	*Lm*+	%	CI 95%	*n*	*Lm*+	%	CI 95%
Equipment	221	24	10.9	7.4–15.7	208	21	10.1	6.7–14.9
Industrial systems	156	22	14.1	9.5–20.4	175	12	6.9	4.0–11.6
Machines	162	16	9.9	6.2–15.4	161	8	5.0	2.5–9.5
Clothing	7	1	14.2	2.6–51.3	7	3	42.9	15.8–75.0
Tools	52	9	17.3	9.4–29.7	65	2	3.1	0.9–10.5
Total	598	72	12.0	9.7–14.9	616	46	7.5	5.7–9.8

* The table does not consider the three samples: the two negative cleaning tools and the not classifiable surface. FPP: food processing plant; RS: retail store; *Lm+*: *Listeria monocytogenes* positive samples.

**Table 4:** cgMLST analysis: number of isolates within cgMLST clusters observed in each clonal complex (CCs) presenting more than one cluster.

CC	cgMLST Clusters	No. of Isolates	No. of Singleton Strains
CC121	6	12	7
		6	
		3	
		2	
		2	
		2	
CC9	6	9	5
		4	
		4	
		3	
		3	
		2	
CC1	3	6	4
		3	
		2	
CC3	2	9	3
		2	

## Data Availability

The genome assemblies were deposited at DDBJ/ENA/GenBank under the BioProject PRJNA737760.

## Supplementary Materials

The following are available online at https://www.mdpi.com/article/10.3390/foods10081944/s1, Figure S1: Minimum Spanning Tree (MST) based on the cgMLST profiles of CC121 *Lm* strains coloured according to sampling session; the cgMLST clusters containing more than two strains are highlighted in red. Figure S2: Minimum Spanning Tree (MST) based on the cgMLST profiles of CC9 *Lm* strains coloured according to sampling session; the cgMLST clusters containing more than two strains are highlighted in red. Figure S3: Minimum Spanning Tree (MST) based on the cgMLST profiles of CC1 *Lm* strains coloured according to sampling session; the cgMLST clusters containing more than two strains are highlighted in red. Figure S4: Minimum Spanning Tree (MST) based on the cgMLST profiles of CC3 *Lm* strains coloured according to sampling session; the cgMLST clusters containing more than two strains are highlighted in red. Figure S5: Minimum Spanning Tree (MST) based on the cgMLST profiles of CC7 *Lm* strains coloured according to sampling session; All the strains belonged to the same cgMLST cluster. Table S1: Abbreviation list; Table S2: List of sampled surfaces grouped into five categories; Table S3: Positive samples and *Lm* strains’ molecular typing results for each production FPP and RS. Table S4: Positive samples and molecular typing results for *Lm* strains isolated during the follow-up sampling; Table S5: Quality control check of sequence data. Reads’ quality control metrics reported are after trimming.
