# Supplementary material for: Intensive Environmental Surveillance Plan for Listeria monocytogenes in Food Producing Plants and Retail Stores of Central Italy: Prevalence and Genetic Diversity
Source: Foods. 2021 Aug 20;10(8):1944. doi: 10.3390/foods10081944 (PMC8392342; doi:10.3390/foods10081944)
Supplement: Supplementary file 1 [file foods-10-01944-s001.zip › figures-supplementary.pdf]

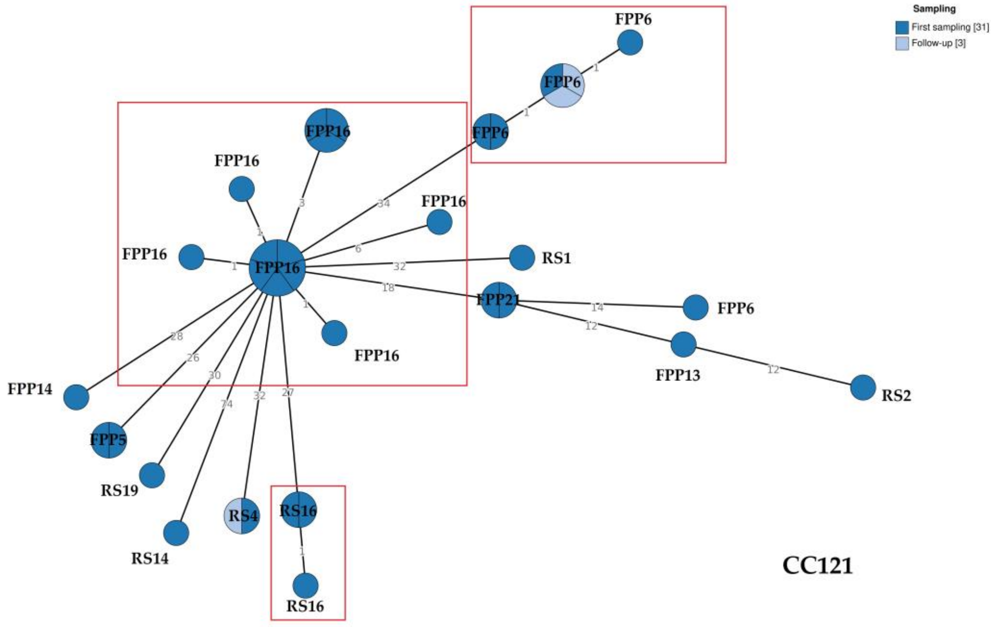

Figure S1. Minimum Spanning Tree (MST) based on the cgMLST profiles of CC121 *Lm* strains coloured according to sampling session; the cgMLST clusters containing more than two strains are highlighted in red.

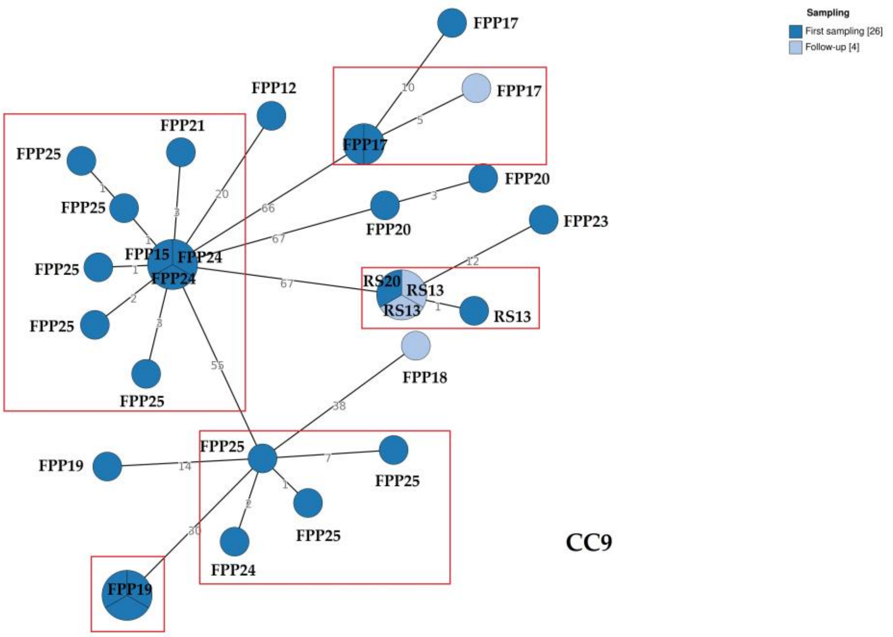

Figure S2. Minimum Spanning Tree (MST) based on the cgMLST profiles of CC9 *Lm* strains coloured according to sampling session; the cgMLST clusters containing more than two strains are highlighted in red.

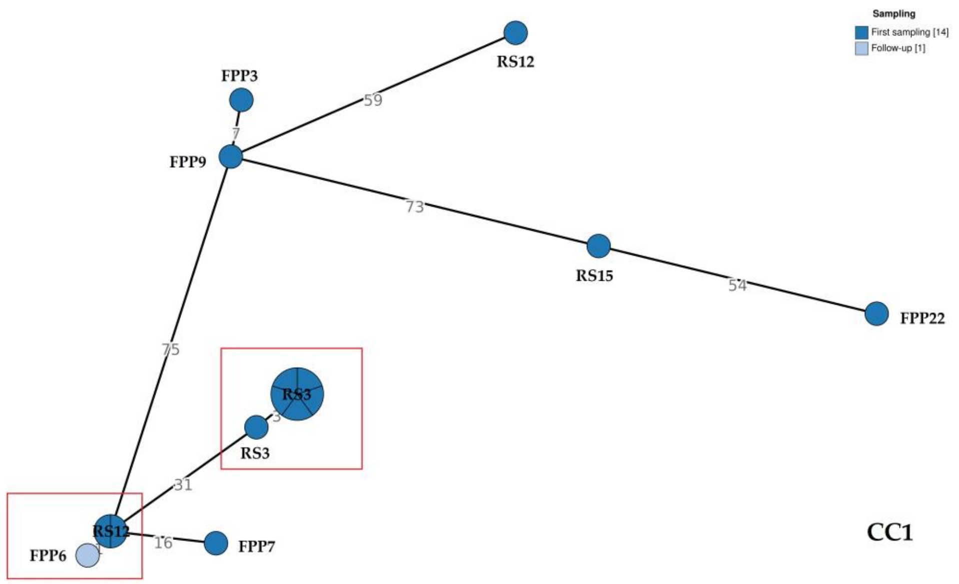

Figure S3. Minimum Spanning Tree (MST) based on the cgMLST profiles of CC1 *Lm* strains coloured according to sampling session; the cgMLST clusters containing more than two strains are highlighted in red.

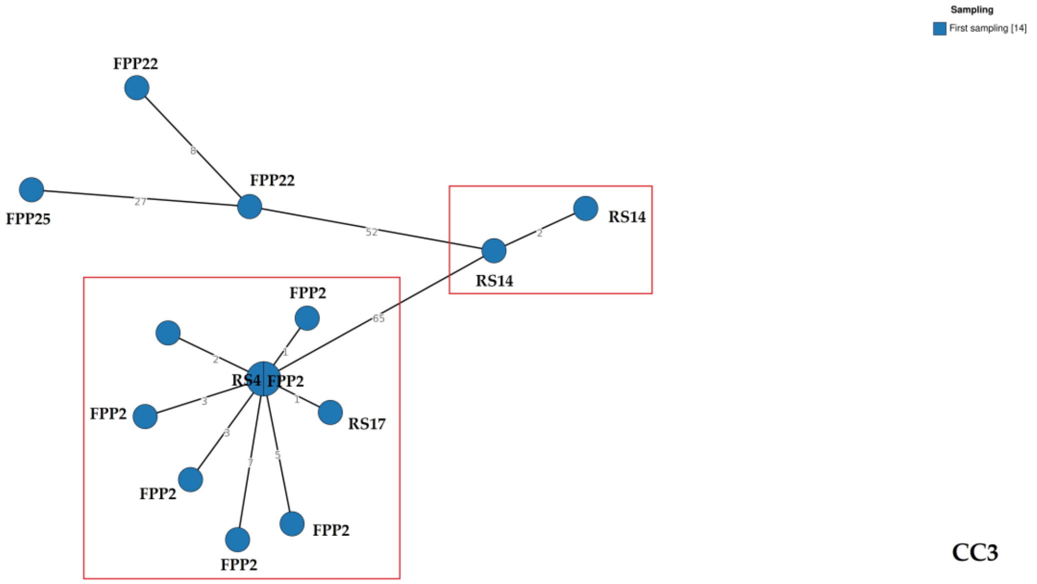

Figure S4. Minimum Spanning Tree (MST) based on the cgMLST profiles of CC3 *Lm* strains coloured according to sampling session; the cgMLST clusters containing more than two strains are highlighted in red.

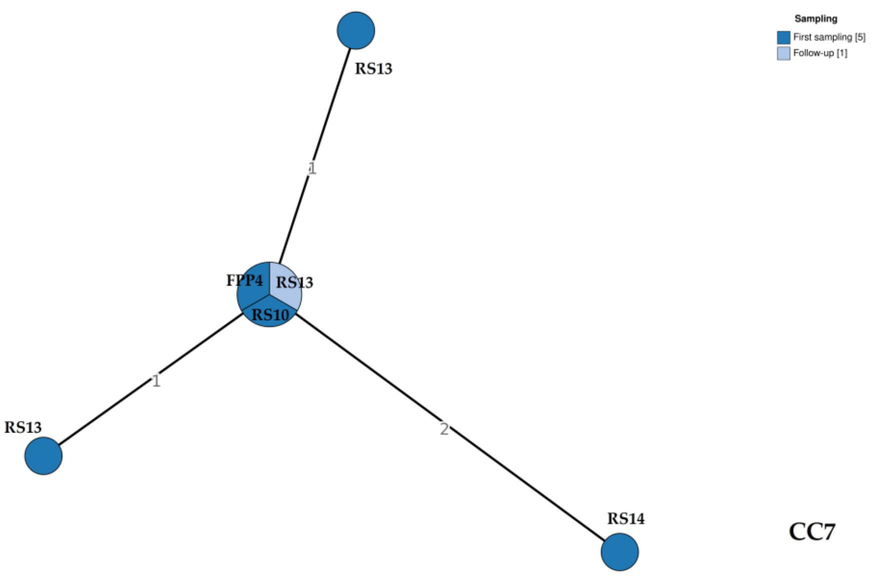

Figure S5. Minimum Spanning Tree (MST) based on the cgMLST profiles of CC7 *Lm* strains coloured according to sampling session; All the strains belonged to the same cgMLST cluster
