# Supplementary material for: Intensive Environmental Surveillance Plan for Listeria monocytogenes in Food Producing Plants and Retail Stores of Central Italy: Prevalence and Genetic Diversity
Source: Foods. 2021 Aug 20;10(8):1944. doi: 10.3390/foods10081944 (PMC8392342; doi:10.3390/foods10081944)
Supplement: Supplementary file 1 [file foods-10-01944-s001.zip › Table_S1.pdf]

Table S1. Abbreviation list.

**Abbreviation**	**Explenation**
FPE	Food processing environment
FPP	Food producing plants
RS	Retail store
FBO	Food business operators
RTE	Ready-to-eat
WGS	Whole genome sequencing
MLST	Multilocus sequencing typing
cgMLST	Core genome multilocus sequencing typing
MST	Minimum spanning tree
