# Supplementary material for: Intensive Environmental Surveillance Plan for Listeria monocytogenes in Food Producing Plants and Retail Stores of Central Italy: Prevalence and Genetic Diversity
Source: Foods. 2021 Aug 20;10(8):1944. doi: 10.3390/foods10081944 (PMC8392342; doi:10.3390/foods10081944)
Supplement: Supplementary file 1 [file foods-10-01944-s001.zip › Table_S2.pdf]

Table S2. List of sampled surfaces grouped into five categories.

Surface category	sampled surfaces
Equipment	industrial cupboards/shelves/drawers
	basins/baskets
	carts
	baldresca/containers
	waste/processing waste containers
	meat cart containers
	meat hanger trolleys/racks for hanging sausages
	hooking bars
	hooks
	cookware
	hams holders
	pushbuttons/keyboards/control panels/switches
	cutting boards
	working/packing tables
	trays/dishes
Industrial systems	air intake systems/cooker hoods
	air conditioning systems
	cooling systems for cold rooms/blast chillers/refrigerators
	cooling systems for refrigerated exhibitors/exhibitor mural refrigerators
	sinks
	drain channels
	drain wells
	cold rooms
	drying rooms
	seasoning rooms
	goods lifts
	walls
	floors
	door/door handles
	food wormers
	pipes
Machines	slicers
	scales
	packaging machines
	meat-bone separators
	labeling machines
	ovens/cooking boilers
	grater machines
	pressure washers
	kneaders
	sausage stuffers
	meat tenderizers
	meat tying machines
	centrifugal fans
	conveyors belts
	bone saws/band saws
	meat injector machines
	knives sterilizers
	meat grinders
Clothing	cut resistant gloves stainless steel
	apron
	gloves
	boots/shoes
Cleaning Tools	water tube
Tools	knife sharpeners
	Can openers
	knives
	spoons/ladles/scoops/strainers
	forks
	tongs
	salami prickers
