# Supplementary material for: Intensive Environmental Surveillance Plan for Listeria monocytogenes in Food Producing Plants and Retail Stores of Central Italy: Prevalence and Genetic Diversity
Source: Foods. 2021 Aug 20;10(8):1944. doi: 10.3390/foods10081944 (PMC8392342; doi:10.3390/foods10081944)
Supplement: Supplementary file 1 [file foods-10-01944-s001.zip › Table_S3_final_proof.pdf]

Table S3. Positive samples and *Lm* strains' molecular typing results for each production FPP and RS.

Food processing plant/retail store P ID	Positive samples (%)[a]	Typed strains	Serogroup	ST	CC
FPP1	6/15 (40%)	6	IIb	191	191
FPP2	7/15 (47%)	7	IIb	3	3
FPP3	1/11 (9%)	1	IVb	1	1
**FPP4**	**1/15 (7%)**	**1**	**IIa**	**7**	**7**
FPP5	2/15 (13%)	2	IIa	121	121
FPP6	5/15 (33%)	5	IIa	121	121
		1	IIb	517	517
FPP7	1/13 (7%)	1	IVb	1	1
FPP8	1/15 /7%)	1	SNT	SNT	SNT
FPP9	1/15 (7%)	1	IVb	1	1
FPP10	2/15 (13%)	2	IIb	517	517
FPP11	1/15 (7%)	1	IVb	6	6
FPP12	1/15 (7%)	1	IIc	9	9
FPP13	1/15 (7%)	1	IIa	121	121
FPP14	1/15 (7%)	1	IIa	121	121
FPP15	1/15 (7%)	1	IIc	9	9
FPP16	12/15 (80%)	12	IIa	121	121
FPP17	3/15 (20%)	3	IIc	9	9
FPP18	2/15 (13%)	1	IIa	14	14
		1	IIb	429	429
FPP19	4/15 (27%)	4	IIc	9	9
FPP20	2/15 (13%)	2	IIc	9	9
FPP21	2/15 (13%)	1	IIc	9	9
		2	IIa	121	121
FPP22	3/15 (20%)	1	IVb	1	1
		2	IIb	3	3
FPP23	1/15 (7%)	1	IIc	9	9
FPP24	3/15 (20%)	3	IIc	9	9
FPP25	8/15 (53%)	1	IIb	3	3
		8	IIc	9	9
RS1	1/15 (7%)	1	IIa	121	121
RS2	2/15 (13%)	1	IVb	1	1
		1	IIa	121	121
RS3	6/15 (40%)	6	IVb	1	1
RS4	5/15 (33%)	1	IIb	3	3
		1	IIa	121	121
		2	IIa	8	8
		1	IIa	101	101
RS5	1/15 (7%)	1	IVb	2	2
RS6	1/15 (7%)	1	IIa	91	14

RS7	1/15 (7%)	1	IIa	8	8
RS8	2/15 (13%)	2	IVb	2	2
RS9	1/7 (14%)	1	IIa	2764	2764
**RS10**	**1/15 (7%)**	**1**	**IIa**	**7**	**7**
RS11	3/12 (25%)	2	IIb	363	363
		1	IIb	224	224
RS12	3/15 (20%)	1	IIa	325	31
		2	IVb	1	1
**RS13**	**3/16 (19%)**	1	IIc	9	9
		**2**	**IIa**	**7**	**7**
RS14	5/15 (33%)	2	IIb	3	3
		1	IIa	7	7
		1	IIa	504	475
		1	IIa	121	121
RS15	1/15 (7%)	1	IVb	1	1
RS16	2/15 (13%)	3	IIa	121	121
RS17	1/15 (7%)	1	IIb	3	3
RS18	1/15 (7%)	1	IIa	325	31
RS19	3/15 (20%)	1	IIa	325	31
		1	IIa	155	155
		1	IIa	121	121
RS20	2/15 (13%)	1	IIa	325	31
		1	IIc	9	9
RS21	1/15 (7%)	1	IIa	325	31

ST-sequence type. CC-clonal complex. a: environmental samples positive for *Lm* compared to the total samples tested. Strains belonging to the outbreak clone are in bold font. SNT- strain not typed: the bacterial strain was not available for further genomic analysis other than serogroup. [UW1]
