# Supplementary material for: Intensive Environmental Surveillance Plan for Listeria monocytogenes in Food Producing Plants and Retail Stores of Central Italy: Prevalence and Genetic Diversity
Source: Foods. 2021 Aug 20;10(8):1944. doi: 10.3390/foods10081944 (PMC8392342; doi:10.3390/foods10081944)
Supplement: Supplementary file 1 [file foods-10-01944-s001.zip › Table_S4.pdf]

Table S4. Positive samples and molecular typing results for *Lm* isolated during the follow-up sampling.

Food processing plant/retail store ID	Typed isolates	Serogroup	ST	CC
FPP6	2	IIa	121	121
	1	IVb	1	1
FPP11	1	IVb	6	6
FPP17	1	IIc	9	9
FPP18	1	IIc	9	9
RS4	1	IIa	121	121
RS6	1	IIa	14	91
**RS13**	1	IVb	2	2
	**1**	**IIa**	**7**	**7**
	2	IIc	9	9

ST-sequence type. CC-clonal complex. Strains belonging to the outbreak clone are in bold font.
