# Supplementary material for: Intensive Environmental Surveillance Plan for Listeria monocytogenes in Food Producing Plants and Retail Stores of Central Italy: Prevalence and Genetic Diversity
Source: Foods. 2021 Aug 20;10(8):1944. doi: 10.3390/foods10081944 (PMC8392342; doi:10.3390/foods10081944)
Supplement: Supplementary file 1 [file foods-10-01944-s001.zip › Table_S5_noAcc.pdf]

Table S5. Quality control check of sequence data. Reads' quality control metrics reported are after trimming.

Strain ID	Sequence ID	Average read quality score	N° read pairs	Vertical coverage	N° contigs	Total length (bp)	N50	L50
Lm_1958	2016.TE.10956.1.10.fsa	32.1	4,013,638	118	65	2,967,331	489,281	3
Lm_1959	2016.TE.10956.1.11.fsa	31.79	2,872,928	92	59	3,017,215	510,825	3
Lm_1960	2016.TE.10956.1.12.fsa	32.7	4,052,512	169	167	2,977,541	524,626	2
Lm_1961	2016.TE.10956.1.13.fsa	31.86	2,182,764	67	52	2,983,122	508,735	2
Lm_1962	2016.TE.10956.1.14.fsa	32.4	2,715,462	115	119	3,025,935	516,315	2
Lm_1963	2016.TE.10956.1.15.fsa	31.97	2,229,538	37	176	3,007,080	489,058	2
Lm_1964	2016.TE.10956.1.16.fsa	32.18	1,945,568	81	82	3,019,088	516,602	2
Lm_1967	2016.TE.10956.1.19.fsa	32.27	3,115,196	133	168	3,028,288	579,539	3
Lm_1968	2016.TE.10956.1.20.fsa	31.79	1,952,728	60	214	2,943,512	48,265	17
Lm_1969	2016.TE.10956.1.25.fsa	32.48	3,931,966	167	159	3,038,424	489,317	3
Lm_1970	2016.TE.10956.1.26.fsa	32.11	4,806,552	137	49	2,935,177	449,383	2
Lm_1974	2016.TE.10956.1.36.fsa	31.61	2,259,980	72	63	2,974,292	268,528	3
Lm_1975	2016.TE.10956.1.37.fsa	32.11	2,255,916	94	95	2,895,712	510,943	2
Lm_1976	2016.TE.10956.1.38.fsa	32.26	2,034,124	85	504	3,096,610	483,057	3
Lm_1977	2016.TE.10956.1.39.fsa	32.18	2,338,262	96	131	3,093,756	431,753	3
Lm_1954	2016.TE.10956.1.40.fsa	32.1	2,270,532	96	81	2,931,423	1,507,844	1
Lm_1948	2016.TE.10956.1.41.fsa	32.25	2,787,522	117	129	3,068,386	526,076	2
Lm_1949	2016.TE.10956.1.42.fsa	32.29	2,498,646	106	145	3,038,436	517,91	2
Lm_1950	2016.TE.10956.1.43.fsa	32.24	2,190,292	91	197	3,109,352	438,891	4
Lm_1951	2016.TE.10956.1.44.fsa	33.46	949,4	31	114	3,065,689	64,672	15
Lm_1952	2016.TE.10956.1.45.fsa	32.39	3,005,852	128	153	3,124,487	481,686	3
Lm_1971	2016.TE.10956.1.46.fsa	32.31	2,519,256	95	75	3,049,803	449,54	4
Lm_1953	2016.TE.10956.1.47.fsa	32.32	2,685,800	114	110	2,924,335	580,305	2
Lm_1978	2016.TE.10956.1.48.fsa	32.23	2,467,484	104	94	2,968,632	479,534	3
Lm_1980	2016.TE.10956.1.49.fsa	32.23	2,307,802	97	112	3,112,802	474,812	3
Lm_1979	2016.TE.10956.1.51.fsa	32.29	2,463,636	105	107	3,025,717	257,394	3
Lm_1957	2016.TE.10956.1.9.fsa	32.09	3,161,934	89	64	3,011,702	507,416	3
2016.TE.12907.1.33	2016.TE.12907.1.33.fsa	34.4	5,305,850	196	53	3,060,351	465,251	4
2016.TE.12907.1.34	2016.TE.12907.1.34.fsa	33.7	2,626,610	117	57	3,061,472	481,622	3
Lm_2001	2016.TE.15639.1.13.fsa	33.06	3,363,790	145	97	3,109,823	358,803	4
Lm_2029	2016.TE.15639.1.17.fsa	33.66	1,440,170	51	67	3,092,990	236,795	5
Lm_2028	2016.TE.15639.1.18.fsa	32.95	3,250,534	142	72	3,098,833	302,516	3
2016.TE.15639.1.26	2016.TE.15639.1.26.fsa	33.48	3,938,672	157	95	2,948,386	449,383	2
Lm_2017	2016.TE.15639.1.28.fsa	33.71	5,612,492	206	116	2,993,205	546,52	3
Lm_2030	2016.TE.15639.1.31.fsa	33.7	2,269,562	81	38	3,008,710	517,925	2
Lm_2032	2016.TE.15639.1.32.fsa	33.73	2,012,494	73	56	3,092,090	302,516	4
Lm_2031	2016.TE.15639.1.33.fsa	33.71	3,892,974	139	58	3,094,072	302,516	4
Lm_2034	2016.TE.15639.1.34.fsa	33.78	3,776,486	132	43	3,053,607	518,041	2

Lm_2042	2016.TE.15639.1.35.fsa	32.94	2,896,316	129	69	3,063,850	510,936	2
Lm_2002	2016.TE.15639.1.5.fsa	33.03	3,842,786	159	85	3,070,489	581,263	2
Lm_2003	2016.TE.15639.1.6.fsa	33.13	2,423,078	105	75	3,103,422	358,803	4
Lm_2033	2016.TE.16633.1.2.fsa	33.83	4,901,730	159	70	3,060,119	518,062	2
Lm_2058	2016.TE.17371.1.10.fsa	34.01	1,392,426	54	61	3,016,064	509,792	3
Lm_2060	2016.TE.17371.1.11.fsa	34.15	1,377,960	47	71	3,012,509	260,686	4
2016.TE.17371.1.18	2016.TE.17371.1.18.fsa	34.17	4,834,256	185	59	3,023,196	518,148	2
Lm_2050	2016.TE.17371.1.2.fsa	34.11	1,218,070	47	66	2,961,586	511,505	3
Lm_2051	2016.TE.17371.1.3.fsa	34.27	4,162,334	146	60	2,928,885	604,997	2
Lm_2052	2016.TE.17371.1.4.fsa	34.08	1,365,764	52	48	2,965,550	489,274	3
Lm_2053	2016.TE.17371.1.5.fsa	34.14	1,378,382	51	46	2,964,998	564,627	3
Lm_2054	2016.TE.17371.1.6.fsa	34.3	4,067,056	142	66	2,976,457	565,717	3
Lm_2055	2016.TE.17371.1.7.fsa	34.17	2,240,044	83	37	3,013,890	511,857	3
Lm_2056	2016.TE.17371.1.8.fsa	34.3	2,647,480	93	41	3,005,661	477,264	3
Lm_2057	2016.TE.17371.1.9.fsa	33.88	3,070,536	131	104	3,023,172	476,54	3
Lm_2091	2016.TE.20838.1.22.fsa	34.03	1,234,212	49	34	3,006,776	476,139	3
Lm_2092	2016.TE.20838.1.23.fsa	33.74	2,416,166	108	75	3,017,832	605,869	2
Lm_2070	2016.TE.20838.1.3.fsa	33.6	3,982,536	167	75	3,029,069	361,235	3
Lm_2069	2016.TE.20838.1.4.fsa	34.23	4,080,212	150	59	3,045,587	435,205	4
Lm_1854	2016.TE.6891.1.78.fsa	33.45	2,067,704	69	53	3,018,896	356,99	2
Lm_1868	2016.TE.6891.1.79.fsa	33.53	1,952,826	68	51	2,935,422	449,383	2
Lm_1936	2016.TE.8594.1.10.fsa	33.32	3,507,926	139	102	3,010,565	294,126	2
Lm_1943	2016.TE.8594.1.11.fsa	33.16	2,222,298	95	60	2,889,692	540,602	2
Lm_1876	2016.TE.8594.1.12.fsa	33.82	3,717,144	137	60	2,985,538	482,817	3
Lm_1877	2016.TE.8594.1.13.fsa	33.26	2,429,142	105	225	3,028,756	482,817	3
Lm_1878	2016.TE.8594.1.14.fsa	33.96	4,800,432	161	121	2,999,846	481,413	3
Lm_1879	2016.TE.8594.1.15.fsa	33.87	3,877,522	142	68	2,972,547	566,621	3
Lm_1880	2016.TE.8594.1.16.fsa	33.89	3,933,478	136	99	3,077,371	479,525	3
Lm_1881	2016.TE.8594.1.17.fsa	33.96	4,363,138	151	47	2,894,234	434,414	3
Lm_1882	2016.TE.8594.1.18.fsa	34.11	3,247,418	99	100	3,086,621	431,753	3
Lm_1883	2016.TE.8594.1.19.fsa	33.6	3,936,462	153	205	3,053,904	505,536	3
Lm_1955	2016.TE.8594.1.2.fsa	34.05	5,651,654	180	77	3,017,824	509,117	3
Lm_1884	2016.TE.8594.1.20.fsa	33.55	3,826,672	154	59	3,014,033	509,119	3
Lm_1885	2016.TE.8594.1.21.fsa	33.19	1,971,426	85	63	3,045,866	429,54	4
Lm_1886	2016.TE.8594.1.22.fsa	33.14	2,419,094	101	59	3,044,510	429,594	4
Lm_1887	2016.TE.8594.1.23.fsa	33.11	3,059,626	128	100	3,055,720	429,594	4
Lm_1888	2016.TE.8594.1.24.fsa	33.82	4,869,248	177	306	3,062,594	357,44	2
Lm_1889	2016.TE.8594.1.25.fsa	33.17	1,909,250	82	52	3,011,401	605,869	2
Lm_1890	2016.TE.8594.1.26.fsa	33.38	2,677,658	111	74	3,036,749	484,925	3
Lm_1891	2016.TE.8594.1.27.fsa	34.08	4,379,378	137	68	3,063,947	484,133	3
Lm_1892	2016.TE.8594.1.28.fsa	33.6	3,735,040	136	57	2,961,573	357,44	2
Lm_1872	2016.TE.8594.1.29.fsa	33.55	3,125,588	101	62	3,018,366	449,477	3
Lm_1956	2016.TE.8594.1.3.fsa	33.62	3,511,986	141	95	3,109,434	358,803	4

Lm_1873	2016.TE.8594.1.30.fsa	34.04	3,041,014	93	64	2,998,313	476,295	3
Lm_1874	2016.TE.8594.1.31.fsa	33.5	3,273,412	128	127	3,116,013	358,802	4
Lm_1875	2016.TE.8594.1.32.fsa	33.94	4,157,440	136	84	3,104,808	482,291	3
Lm_1900	2016.TE.8594.1.33.fsa	33.66	3,168,604	121	89	3,100,462	358,802	4
Lm_1901	2016.TE.8594.1.34.fsa	33.6	3,540,108	132	620	3,244,089	358,802	4
Lm_1902	2016.TE.8594.1.35.fsa	33.25	2,813,316	119	49	2,908,723	580,306	2
Lm_1903	2016.TE.8594.1.36.fsa	33.81	4,710,798	168	84	2,990,627	540,999	2
Lm_1904	2016.TE.8594.1.37.fsa	33.12	1,991,218	84	67	2,967,112	299,173	2
Lm_1905	2016.TE.8594.1.38.fsa	33.49	2,650,140	103	167	3,044,341	356,99	2
Lm_1906	2016.TE.8594.1.39.fsa	33.92	3,456,078	118	72	3,019,502	356,99	2
Lm_1907	2016.TE.8594.1.40.fsa	33.93	3,246,432	103	70	3,018,019	356,99	2
Lm_1908	2016.TE.8594.1.41.fsa	33.86	1,917,054	63	124	3,031,225	356,99	3
Lm_1909	2016.TE.8594.1.42.fsa	33.91	2,627,260	91	113	3,068,187	376,529	2
Lm_1910	2016.TE.8594.1.43.fsa	33.37	2,939,254	117	71	3,020,560	356,989	3
Lm_1911	2016.TE.8594.1.44.fsa	34.09	5,225,150	158	101	3,086,604	431,753	3
Lm_1912	2016.TE.8594.1.45.fsa	33.41	1,823,574	77	454	3,198,918	358,803	4
Lm_1913	2016.TE.8594.1.46.fsa	33.91	5,523,864	191	96	2,983,671	295,975	4
Lm_1914	2016.TE.8594.1.47.fsa	33.39	2,928,668	123	76	2,979,956	308,368	4
Lm_1918	2016.TE.8594.1.48.fsa	33.68	4,600,160	181	116	3,076,932	482,727	3
2016.TE.8594.1.5	2016.TE.8594.1.5.fsa	34.11	3,919,570	126	40	2,932,597	437,349	2
Lm_1920	2016.TE.8594.1.50.fsa	33.39	2,292,424	96	723	3,244,126	344,532	4
Lm_1921	2016.TE.8594.1.51.fsa	34.12	3,824,204	124	55	3,061,470	482,464	3
Lm_1922	2016.TE.8594.1.52.fsa	34.03	4,307,648	142	81	3,067,703	481,06	3
Lm_1924	2016.TE.8594.1.54.fsa	33.98	2,980,650	103	57	3,061,073	465,251	3
Lm_1926	2016.TE.8594.1.56.fsa	34.1	5,039,372	160	127	3,078,656	344,262	4
Lm_1927	2016.TE.8594.1.57.fsa	33.36	1,906,334	82	64	3,062,541	358,322	4
Lm_1928	2016.TE.8594.1.58.fsa	34.03	5,004,246	165	45	3,059,159	482,727	3
Lm_1930	2016.TE.8594.1.59.fsa	33.38	2,973,294	126	141	3,034,201	564,718	3
Lm_1931	2016.TE.8594.1.60.fsa	33.43	3,335,494	136	119	3,113,267	431,753	3
Lm_1933	2016.TE.8594.1.61.fsa	33.34	1,862,716	77	64	3,042,551	294,126	2
Lm_1934	2016.TE.8594.1.62.fsa	32.89	2,101,804	83	154	3,067,070	294,126	3
Lm_1935	2016.TE.8594.1.63.fsa	34.05	4,107,590	125	61	3,000,428	294,126	2
Lm_1937	2016.TE.8594.1.64.fsa	34.04	3,740,640	112	63	3,042,414	294,126	2
Lm_1938	2016.TE.8594.1.65.fsa	34.01	3,242,648	103	77	2,974,254	262,424	2
Lm_1939	2016.TE.8594.1.66.fsa	34.15	3,032,410	95	82	3,074,215	481,38	3
Lm_1940	2016.TE.8594.1.67.fsa	33.18	2,350,758	97	92	3,107,778	307,644	4
Lm_1941	2016.TE.8594.1.68.fsa	33.98	4,343,402	142	61	3,100,318	482,801	3
Lm_1942	2016.TE.8594.1.69.fsa	33.6	2,499,532	96	64	3,100,133	482,429	3
2016.TE.8594.1.70	2016.TE.8594.1.70.fsa	33.69	3,898,756	147	207	3,161,174	358,181	4
Lm_1944	2016.TE.8594.1.71.fsa	33.45	3,234,282	130	157	3,117,503	431,752	3
Lm_1945	2016.TE.8594.1.72.fsa	33.25	2,101,106	83	113	3,075,044	356,99	2
Lm_1946	2016.TE.8594.1.73.fsa	33.18	2,057,446	83	134	3,080,721	357,182	2
Lm_1917	2016.TE.8594.1.74.fsa	33.25	2,284,278	98	105	3,109,227	482,505	3

Lm_1947	2016.TE.8594.1.9.fsa	33.2	1,621,580	65	53	3,039,033	261,325	2
Lm_1981	2016.TE.9198.1.24.fsa	33.15	1,272,262	44	28	2,992,761	262,448	3
Lm_1982	2016.TE.9198.1.25.fsa	32.97	1,500,928	52	62	3,001,267	262,447	2
2020.TE.88968.1.2	2020.TE.88968.1.2.fsa	36.8	1,513,740	75	42	3,100,088	482,067	3
Lm_1923	2020.TE.88968.1.3.fsa	36.77	1,268,386	63	29	3,056,186	465,338	3
Lm_1973	2020.TE.88968.1.4.fsa	36.78	1,452,784	72	21	2,846,401	1,464,595	1
2020.TE.88968.1.5	2020.TE.88968.1.5.fsa	36.78	1,250,626	62	47	3,098,667	480,32	3
2020.TE.88968.1.6	2020.TE.88968.1.6.fsa	36.78	1,736,240	86	46	3,099,491	482,504	3
Lm_1999	2020.TE.88968.1.7.fsa	36.78	1,238,390	61	48	3,098,775	480,726	3
